# Evaluating the new family medicine internship programmes in the Western Cape, South Africa

**DOI:** 10.4102/safp.v66i1.5837

**Published:** 2024-05-10

**Authors:** Lauren N. Hutton, Louis S. Jenkins, Robert Mash, Klaus von Pressentin, Steve Reid, Jennie Morgan, Paul Kapp

**Affiliations:** 1 Department of Family and Emergency Medicine, Division of Family Medicine and Primary Care, Faculty of Medicine and Health Sciences, Stellenbosch University, Cape Town, South Africa; 2 Knysna Hospital, Garden Route District, Western Cape Department of Health, Knysna, South Africa; 3 Department of Family Medicine and Primary Care, Faculty of Health, Stellenbosch University, Cape Town, South Africa; 4 Primary Health Care Directorate, Department of Family, Community and Emergency Care, Faculty of Health Sciences, University of Cape Town, Cape Town, South Africa; 5 George Hospital, Garden Route District, Western Cape Department of Health, George, South Africa; 6 Department of Family, Community and Emergency Care, Faculty of Health Sciences, University of Cape Town, Cape Town, South Africa

**Keywords:** interns, internship, family medicine, primary care, primary health care, clinical training, medical education

## Abstract

**Background:**

In 2021, South Africa introduced a new 6-month internship in family medicine and primary care. This study aimed to assess the new rotation at district health facilities in the Western Cape.

**Methods:**

A descriptive survey of interns and supervisors, as phase-two of an exploratory sequential mixed methods study. Questionnaires were developed from a descriptive exploratory qualitative study. Data were analysed with the Statistical Package for Social Sciences.

**Results:**

Questionnaires were completed by 72 interns (response rate 21%) and 36 supervisors (response rate 90%), across 10 training programmes. Interns felt more independent (97.2%), confident (90.3%) and resilient (91.6%). They learnt to manage undifferentiated and chronic conditions (91.6%), to refer patients (94.3%) and conduct procedures (77.8%). Most interns were not exposed to community-based services (68.0%) and continuity of care (54.1%). Supervision was mostly adequate during the day (79.1%) and afterhours (80.6%). Many interns reported no structured teaching programme (41.7% – 55.6%). Most supervision was from medical officers and registrars. Supervisors saw interns as valuable members of the clinical team (100.0%), who required extra support and administration (42.5%). The majority of interns (75.0%) and supervisors (72.7%) thought the rotation was the right length and the best preparation for community service (67.6%).

**Conclusion:**

The rotation met most expectations of the Health Professions Council of South Africa. Programmes need to improve exposure to community-orientated primary care, public health medicine, palliative and ongoing care. Supervision and orientation of interns needs improvement.

**Contribution:**

This is the first evaluation of the new family medicine internship programme in South Africa.

## Background

A medical intern is a doctor in training, who has completed their undergraduate degree and is working as a junior doctor through apprenticeship in a specified internship programme. The Health Professions Council of South Africa (HPCSA) is responsible for determining the required training length and type. The purpose of the internship is for newly qualified doctors to practise under supervision in a training post so that they are safe and competent to practise independently.^1^

Medical internship programmes differ across the globe. However, satisfactory completion of internship is a requirement for medical graduates to obtain a full licence to practise in all countries. The United Kingdom has a 2-year foundation programme, with graduates working in different areas of the National Health Service.^2^ In Australia and New Zealand, a 1-year internship must be completed, divided into four rotations to cover the major medical specialities.^3^ The United States differs, whereby the first year of a chosen 3–7-year residency is equivalent to an internship.^4^

In South Africa (SA), the guidelines for medical internship have evolved over the past 70 years. Starting from the 1950s, internship consisted of one practical year of experience, to consolidate undergraduate learning.^5^ Initially, the clinical disciplines experienced during this year were chosen by the intern, under guidelines from the South African Medical and Dental Council and hospital managers. A 1986 study evaluated the internship year in SA and found that the disciplines of internal medicine, surgery, obstetrics and gynaecology, and paediatrics were the most favoured, with only 1% of interns showing an interest in community health, as it was defined at the time.^6^ However, it was not possible to do internship in community health prior to the changes to the internship in 2004.

In July 2004, the internship was extended to 2 years, when the HPCSA created new guidelines for doctors in training. This was adopted, because of reports that newly graduated doctors lacked clinical skills, particularly in obstetrics and anaesthetics at district hospitals (DHs).^7^ At that time there was also an intention by the HPCSA to reduce undergraduate training from 6 years to 5 years, which eventually did not materialise.^8^ These 2 years of internship were structured to involve 4-month rotations through the major specialities (medicine, surgery, obstetrics and gynaecology, paediatrics), 3-months of family medicine, 2-months of orthopaedics and anaesthetics, and 1-month of psychiatry.

The full 2 years were spent within various specialities at an accredited hospital, typically a secondary or tertiary hospital. There was some exposure to primary and district healthcare in the 3-month family medicine rotation, at facilities linked to the training hospital. This was the first time that interns were required to have some exposure to family medicine and was in line with a growing global understanding of the need for and value of medical generalism, particularly for the training of doctors in undifferentiated and chronic conditions in the community.^9^

The most recent change to South Africa’s medical internship programme was in 2021 with the introduction of a 6-month rotation in family medicine during the second year of training.^1^ These 6 months are spent at DHs and primary health care (PHC) facilities, led by family physicians. The HPCSA sets specific aims and objectives for learning, with a logbook to clarify and document the required knowledge, skills and experience. The HPCSA logbook is the guiding tool for interns to record what was learned and track progress throughout the 6-month rotation, with assessments at 2, 4 and 6 months.^10^ In terms of the key learning outcomes and requirements, as per the HPCSA logbook for the family medicine rotation, the rotation aimed to produce a generalist doctor, who at the end of internship will:

(a) have the knowledge and skills to function at a district hospital, with necessary access to supervision, support and referral systems; (b) have the ability to work independently in ambulatory care, within the district health system; (c) be able to manage all patients presenting for care at primary care facilities; and (d) apply knowledge, skills and attitudes in the management of these patients, while collaborating with other staff and referral centres.^11^

Medical managers, clinical managers, and intern curators at accredited facilities are responsible for ensuring that the HPCSA’s requirements are met.

In SA, doctors are obligated to complete a year of community service after their internship. They are placed anywhere in the country by the Department of Health, according to the need, often in the district health services. It is expected that the internship programme should prepare doctors for community service, where they may have minimal supervision in under-resourced health facilities. One study in 2013, from KwaZulu-Natal, suggested that community service doctors still had critical skills gaps, several of which could be addressed by additional exposure to family medicine and primary care in the internship programme.^12^

As of 2021, the first year was divided into 3-month rotations through internal medicine, surgery, paediatrics and obstetrics. This year is standard for all interns across the country. All interns then spend 6-months of the second year in a family medicine and primary care rotation, and the remainder of the year rotating for 2 months through each of orthopaedics, psychiatry and anaesthesia. This rotation, with increased exposure to family medicine and psychiatry, was thought by HPCSA to better prepare interns for community service.

Despite all these changes to the structure of the intern programme, there has been little research to evaluate the effects that these have had on community service or junior doctors.

The National 2030 Human Resources for Health strategy has, as one of its goals, the desire to build ‘an enabled, productive, motivated and empowered health workforce’, to ensure a functional district health system and progress towards universal health coverage.^11^ It calls for a critical review of the internship and community service policies. Evaluating the family medicine component of the new internship programme will contribute to such a review.

Although the HPCSA has defined standard outcomes for the family medicine rotation, there is a significant variation in how internship training complexes have organised their rotations. As interns complete this new rotation for the first time, it is important to evaluate whether the outcomes have been achieved, how interns and supervisors experienced the rotation, what they learnt and how the variety of models for organising the rotation compare.

The study aimed to assess the new 6-month family medicine rotation for medical interns at district health facilities in the Western Cape. Specific objectives were to evaluate the learning of interns and how they developed as health professionals and to identify the strengths and weaknesses of the programme as well as the perceived impact that interns had on the healthcare service. Insights gained from this study will help shape the intern programme in the Western Cape with possible transferable lessons for other South African provinces.

## Methods

### Study design

This was a cross-sectional descriptive observational survey of interns and their supervisors. The survey was the second phase of an exploratory mixed methods study that had an exploratory qualitative study in the first phase.^13^ The themes from this first study informed the design of the questionnaire for the survey.

### Study setting

The Western Cape province accommodates medical interns in both the metro health services (MHS) and rural health services (RHS). The public health services in this province consist of three tertiary facilities, five regional hospitals and 37 DHs. Intern programmes are based at tertiary, regional and large (more than 100 beds) DHs. The surrounding smaller DHs and primary care facilities are used for the family medicine training programme. In 2020, there were 660 internship posts, across 10 training complexes in the province, with half of the interns in their first year and half in their second year. These complexes utilised 13 DHs and 38 PHC facilities for the family medicine programme in the second year.

### Study population, sample size and sampling

The study population was all 330 second-year interns and their supervisors involved in family medicine intern training at Western Cape facilities in 2022. Supervisors included clinical trainers in the workplace, managers of health facilities where the interns were placed, and curators, who were responsible for coordinating the whole rotation on behalf of the base hospital. An estimate of 40 supervisors was made based on two clinical trainers, one facility manager and one curator for each of the 10 training complexes. There was no sampling and the whole study population was invited to participate.

### Data collection

Two questionnaires were designed, one for the interns and one for the supervisors, based on the themes identified in the qualitative study.^13^ The questionnaires were designed in English by the principal researcher and their content was validated by the authors who were also involved in the qualitative study. The questionnaires were piloted with three interns and one supervisor prior to use, to ensure face validity and feasibility, and appropriate adaptations were made. These data were not included in the study.

Questionnaires were created in REDCap (computer program). version 14.0.7. Nashville: Vanderbilt University; 2024, and administered electronically to respondents. An electronic link, sent via email, was used to distribute the questionnaires. Email addresses were obtained by supervisors and intern representatives. The questionnaires consisted of two types of questions: those with a Likert scale of four options (strongly agree, agree, disagree, strongly disagree) and those with a list of options where ranking was required. Surveys were distributed in the second half of the year, over a period of 3 months. Family physician supervisors and intern curators assisted with accessing the interns and supervisors. An automatic reminder was sent every week to those who started but did not complete the survey.

### Data analysis

Data were exported from REDCap into the SPSS Statistics for Windows (computer program). Version 29.0. Armonk, NY: IBM Corp and analysed by the principal researcher with support from the research team. Categorical data were described as frequencies and percentages. Numerical data were described as means and standard deviations or medians and interquartile ranges depending on the distribution of the data.

### Ethical considerations

Ethical approval for the study was obtained from the Health Research Ethics Committees at Stellenbosch University (Ref: N21/06/053) and the University of Cape Town (Ref: 410/2021). Permission was obtained from the Western Cape Department of Health (Ref: WC_202108_013). Consent was obtained within the questionnaire. Data were stored confidentially using REDcap, with only the authors having access.

## Results

Overall, 72 interns completed the questionnaire of whom 58.3% were female (*n* = 42/72) and 40.3% were male (*n* = 29/72). The response rate was 21.8% (*n* = 72/330). The median age was 26 years (interquartile range [IQR]: 26–27). They came from 10 different internship programmes, as shown in Table 1.

**TABLE 1:** Location of interns (*n* = 72) and supervisors (*n* = 36).

Programme	Interns		Supervisors	
	*n*	%	*n*	%
George	12	16.7	8	22.2
Groote Schuur	26	36.1	12	33.3
Helderberg	6	8.3	2	5.6
Karl Bremer	1	1.4	0	0.0
Mitchells Plain	6	8.3	5	13.9
New Somerset	3	4.2	2	5.6
Paarl	4	5.6	2	5.6
Tygerberg	11	15.3	2	5.6
Victoria	3	4.2	2	5.6
Worcester	0	0.0	1	2.8

In addition, 36 supervisors completed the questionnaire. This represents an estimated response rate of 90%. Of the supervisors, 24 (66.7%) were men and 12 (33.3%) were women, with a median age of 42.5 years (IQR: 39.0–52.0). They were also spread across 10 different programmes (Table 1). Most were based at DHs (55.6%, *n* = 20), followed by PHC (27.8%, *n* = 10), regional hospitals (13.9%, *n* = 5) and a specialist hospital (2.8%, *n* = 1). Most supervisors were clinical trainers (72.2%, *n* = 26), followed by hospital managers (16.7%, *n* = 6) and intern curators (11.1%, *n* = 4). Their job titles included family physicians (44.4%, *n* = 16), clinical managers (22.2%, *n* = 8), medical officers (16.7%, *n* = 6), heads of clinical units (5.6%, *n* = 2) and other specialists (11.1%, *n* = 4).

Table 2 reports on the learning experience of the interns and the proportion of respondents who agreed with the given statements. Interns and supervisors agreed that interns took more independent responsibility for patient care (97.2% and 97.0%), and interns gained confidence in clinical decision-making (90.3%). Interns felt that they increased their resilience and ability to cope with the workload (91.6%). During the rotation they learnt to manage a variety of new as well as undifferentiated conditions (91.6%), and the management of people with chronic conditions (91.6%). In addition, they learnt how to coordinate care between PHC, DH and other referral hospitals (94.3%). They were also able to learn new procedural skills (75.0%) and practise existing ones (77.8%), although supervisors appeared to be more certain about this (93.9% and 100.0%) than the interns. The interns felt that they learnt a more holistic approach to the patient care (84.7%), although many did not learn to consider the family and community context (44.5%). The majority (73.6%) also had the opportunity to engage in activities to improve the quality of care and patient safety. Most interns, however, were not exposed to community-based services (including hospice or palliative care) (68.0%) and did not experience continuity of care with patients (54.1%).

**TABLE 2:** Learning of interns during the family medicine rotation.

Statements	Agree		Strongly agree		Total	
	*n*	%	*n*	%	*n*	%
**Responses from interns (*n* = 72)**						
I took more responsibility for patient care and clinical decision-making in this rotation.	32	44.4	38	52.8	70	97.2
I learned how to refer a patient from the district health services to regional/tertiary hospitals.	33	45.8	35	48.6	68	94.4
I learned how to coordinate care between primary care and the district hospital.	52	73.2	15	21.1	67	94.3
I learned how to assess patients who presented with an undifferentiated problem.	53	73.6	13	18.0	66	91.6
I learned how to provide ongoing care for people with chronic conditions.	33	45.8	33	45.8	66	91.6
I learned how to develop my own resilience and cope with the workload.	50	69.4	16	22.2	66	91.6
I gained confidence in dealing with common presenting problems in primary care.	44	61.1	21	29.2	65	90.3
I learned how to manage a variety of new conditions.	37	51.4	26	36.1	63	87.5
During this rotation, I was able to put into practice all that I have learned in the other rotations.	41	56.9	20	27.8	61	84.7
I learned to consider why the patient had come for help now and their perspective on the illness.	48	66.7	13	18.1	61	84.7
I was able to gain more experience in certain procedures.	40	55.6	16	22.2	56	77.8
I learned how to perform a number of new procedures.	35	48.6	19	26.4	54	75.0
I was able to participate in activities to improve the quality of care and patient safety.	42	58.3	11	15.3	53	73.6
I managed to rotate to all facilities in the sub-district [*PHC, EC, wards, OPD and theatre*].	38	52.8	14	19.4	52	72.2
I spent very little time in the community, NGOs, hospice, PHC or home visits.	25	34.7	24	33.3	49	68.0
I learned how to coordinate care with community health worker teams in the community.	40	55.6	8	11.1	48	66.7
I learned to consider the family and community context more when assessing patients.	42	33.3	16	22.2	58	55.5
I was able to see the same patient over time and have some continuity of care.	31	43.1	2	2.8	33	45.9
I was able to provide care in the community.	21	29.2	4	5.6	25	34.8
There was too much focus on emergency care and not enough exposure to chronic conditions.	6	4.8	2	2.8	8	7.6
**Responses from supervisors (*n* = 33)**						
Interns learned how to manage a variety of new acute conditions.	15	45.5	18	54.5	33	100.0
Interns were able to enhance current skills for performing known procedures.	23	69.7	10	30.3	33	100.0
Interns took responsibility for patient care and decision-making in this rotation.	22	66.7	10	30.3	32	97.0
Interns learned how to manage chronic disease patients at clinic level.	19	57.6	12	36.4	31	93.9
Clinical and personal growth was clearly visible across the 6-month rotation.	18	54.5	13	39.4	31	93.9
Interns learned how to perform a number of new procedures.	23	69.7	8	24.2	31	93.9
Interns were exposed to community health care and involvement.	16	48.5	6	18.2	22	66.7
It was easy to rotate interns equally between PHC, EC, theatre and maternity.	17	51.5	3	9.1	20	60.6
Interns had sufficient exposure to hospice and home-based care.	8	25.0	0	0.0	8	25.0
Interns spent too little time in PHC.	7	21.2	1	3.0	8	24.2

PHC, primary health care; EC, emergency centre; OPD, outpatient department; NGO, non-governmental organisations.

Table 3 reports on use of the HPCSA logbook and the proportion of respondents that agreed with the various statements. The supervisors mostly felt that the logbook did guide the training of the interns (81.8%); however, around half (51.5%) felt that it was not timeously completed throughout the rotation. While many interns reported that the logbook guided them in their learning (59.1%) and helped assess progress (56.3%), over 40% of interns did not feel this way.

**TABLE 3:** Feedback on the Health Professions Council of South Africa logbook.

Statements	Agree		Strongly agree		Total	
	*n*	%	*n*	%	*n*	%
**Intern responses (*n* = 71)**						
The HPCSA logbook guided me in what I should be learning during the rotation.	38	53.5	4	5.6	42	59.1
The HPCSA logbook helped me assess my progress during the rotation.	36	50.7	4	5.6	40	56.3
What I actually learned was well aligned with the outcomes written in the HPCSA logbook.	44	62.0	4	5.6	48	67.6
**Supervisor responses (*n* = 33)**						
The HPCSA logbook guided training of the interns.	26	78.8	1	3.0	27	81.8
The HPCSA logbook was not completed timeously throughout the rotation.	16	48.5	1	3.0	17	51.5

HPCSA, Health Professions Council of South Africa.

Table 4 reports on supervision during the rotation and the proportion of respondents who agreed with the various statements. Although most interns had adequate supervision during the day (79.1%) and after hours (80.6%), approximately one in five interns felt it was insufficient. Many supervisors (36.3%) also reported struggling with providing adequate supervision, particularly in the PHC facilities. Supervisors believed that interns were well-orientated at the beginning of the rotations (97.0%), while only 73.7% of interns agreed with them. Many interns reported that there was no structured teaching programme at the DHs (41.7%) or PHC facilities (55.6%). Supervisors were more likely to report a structured teaching programme (84.8%).

**TABLE 4:** Supervision and teaching opportunities.

Statements	Agree		Strongly agree		Total	
	*n*	%	*n*	%	*n*	%
**Intern responses (*n* = 72)**						
The intern supervisors were approachable and helpful during the rotation.	42	58.3	17	23.6	59	81.9
I had adequate supervision after hours, which enabled me to learn without risking harm to patients or myself.	48	66.7	10	13.9	58	80.6
I had adequate supervision during the day, which enabled me to learn without risking harm to patients or myself.	43	59.7	14	19.4	57	79.1
I was orientated through a structured programme to understand what was expected of me.	49	68.1	4	5.6	53	73.7
The facilities where I worked had an established and supportive culture of learning.	39	54.2	12	16.7	51	70.9
The intern curator was approachable and helpful during the rotation.	33	45.8	17	23.6	50	69.4
There was a structured programme of teaching for the interns at the RH.	38	52.8	7	9.7	45	62.5
There was a structured programme of teaching for the interns at the DH.	32	44.4	10	13.9	42	58.3
I was able to join in the continuing professional development programme with other staff.	35	48.6	6	8.3	41	56.9
There was a structured programme of teaching for the interns at the PHC clinics.	24	33.3	8	11.1	32	44.4
A mentorship programme for junior doctors was present at my facility.	19	26.4	7	9.7	26	36.1
I was left alone at the primary healthcare level with inadequate supervision.	10	13.9	5	6.9	15	20.8
**Supervisor responses (*n* = 33)**						
Interns were well-orientated at the start of their rotation.	21	63.7	11	33.3	32	97.0
Interns were always supervised during daytime hours in the hospital.	16	48.5	16	48.5	32	97.0
There was a structured teaching platform for interns to join.	20	60.6	8	24.2	28	84.8
Interns were always supervised during after-hour activities.	18	54.5	9	27.3	27	81.8
Interns were always supervised in primary healthcare.	18	54.5	7	21.2	25	75.8
The interns were responsible for participation in CPD and learning activities.	18	54.5	6	18.2	24	72.7
A mentorship programme exists for interns at my hospital.	15	45.5	3	9.1	18	54.6
It was a challenge to always provide supervision for interns.	11	33.3	1	3.0	12	36.3

RH, regional hospital; DH, district hospital; PHC, primary health care; CPD, continued professional development.

Just over half (56.9%) of the interns were able to join a continuing professional development (CPD) programme at the facility. Supervisors were felt to be approachable and helpful (81.9%), while intern curators less so (69.4%). Interns reported that they received the most clinical support from medical officers, including community service doctors, and registrars. Family physicians were less available and ranked fourth along with nurses as a source of clinical support.

Table 5 reports on the impact of interns on service delivery and the proportion of the respondents who agreed with the various statements. Supervisors saw interns as valuable members of the clinical team (100.0%), who made a positive improvement to service delivery (86.1%). Interns felt integrated into the clinical teams as trusted members (93.0%). They were not experienced as a burden (93.9%), although they required extra support and administration (42.5%).

**TABLE 5:** Impact on service delivery and overall feedback on the rotation.

Statements	Agree		Strongly agree		Total	
	*n*	%	*n*	%	*n*	%
**Intern responses (*n* = 72)**						
My clinical assessments and opinions were trusted by my senior colleagues.	51	70.8	16	22.2	67	93.0
My intern colleagues and I made a clear difference to health services.	46	64.8	19	26.8	65	91.6
I recognized the value of family medicine as a speciality.	36	50.7	27	38.0	63	88.7
I was acknowledged and included as a full member of the clinical team.	34	47.9	26	36.6	60	84.5
The district hospital/PHC environment is more conducive to fostering independence in decision-making compared to the specialist hospital environment.	40	56.3	20	28.2	60	84.5
The duration of the family medicine rotation was just right.	25	34.7	29	40.3	54	75.0
The family medicine rotation is the best rotation in preparation for the community service year.	25	35.2	23	32.4	48	67.6
I felt more connected with the clinical team in the district hospital/PHC environment compared to the specialist hospital environment.	25	35.2	17	23.9	42	59.1
The family medicine rotation was my favourite rotation during my internship.	22	31.0	8	11.3	30	42.3
I was just an extra pair of hands, used to fill gaps.	12	9.5	6	8.5	18	18.0
**Supervisor responses (*n* = 33)**						
Interns were valuable members of the clinical team.	13	39.4	20	60.6	33	100.0
The duration of the rotation was just right.	8	24.2	16	48.5	24	72.7
The interns were resilient and able to cope with their workload.	22	66.7	1	3.0	23	69.7
Interns added extra administrative work for managers and supervisors.	12	36.4	2	6.1	14	42.5
Interns were inexperienced and needed a lot of extra help when on duty.	12	36.4	0	0.0	12	36.4
The interns had difficulty making clinical decisions and negatively impacted service delivery.	5	13.9	0	0.0	5	13.9
Interns were seen as a burden to senior MOs and COSMOs.	2	6.1	0	0.0	2	6.1

PHC, primary health care; MOs, medical officers; COSMOs, community service medical officers.

Most interns (75.0%, *n* = 54/72) and supervisors (72.7%, *n* = 24/33) felt the rotation was the right length, with some interns feeling it was too long (19.4%, *n* = 14/72) and some supervisors feeling it was too short (21.2%, *n* = 7/33). Most interns reported that the rotation helped them recognise the speciality of family medicine (88.7%), and two-thirds (67.6%) thought this rotation was the best to prepare them for community service.

Interns ranked the most important aspects of the rotation as: (1) increased confidence, (2) developing an approach to undifferentiated patients, (3) personal growth, (4) learning clinical procedures, (5) understanding how the district health system works, (6) making referrals and (7) inter-professional teamwork.

## Discussion

Most interns reported that the family medicine and primary care rotation was the best rotation to prepare them for community service and the majority thought that 6 months was the right length. Many of the HPCSA’s expected outcomes were met,^1^ particularly the management of undifferentiated and chronic conditions, opportunities to practise procedural and surgical skills, and to learn how to refer patients between levels of care. Interns were exposed to ambulatory and emergency care and to both PHC and DH care.^13^ Time spent in the different clinical areas depended on the site. Interns gained confidence, felt more independent and developed resilience to work in resource-constrained settings with high workloads. They appreciated the need for a more holistic approach that considered the patient’s perspective.

They also had the opportunity to integrate and revisit skills that they learnt in the first year of the programme. Overall, the key findings in this survey resonated with the qualitative findings that were previously reported.^12^

Most interns did not experience continuity of care and ‘personal follow up of patients previously seen’.^1^ This could be due to short exposures to different settings during the 6-months and because continuity of care is a weakness of the health services in general.^14^ Although the HPCSA listed ‘appropriate skills in palliative care’ as an outcome,^1^ this was not achieved by the majority of interns. Interns appeared to become more holistic, but mainly in terms of considering the patient’s perspective and reasons for the encounter, rather than their family and community context. Although interns reported on the value of inter-professional teamwork in the qualitative study, this was not ranked highly as a key area of learning.^13^

Very few interns engaged with community-orientated primary care (COPC), community health workers or community-based services. This reflects the focus of clinicians on facility-based services and the need to improve the implementation of COPC throughout the Western Cape.^15^ From 2023, COPC is the official model of care throughout the province and the opportunities for learning should improve.^16^ Although the majority of interns reported exposure to quality improvement activities, the impression was that many of the listed public health medicine objectives were not met,^1,13^ for example, making a community diagnosis, promoting health in communities, evaluation of a health intervention, planning disease prevention and describing the main health indicators. Likewise, there was no mention of the HPCSA’s recommendation that a public health specialist be available to guide learning on these matters.^13^

Most clinical support and supervision were from medical officers and registrars. This cadre of staff would be most accessible and acceptable to the interns. Community service medical officers were listed as the second commonest source of clinical support, which goes against the requirement of the HPCSA for supervision by a ‘general medical practitioner with at least 3-years post-internship experience’.^1^ Again, this may reflect the ease of access to such doctors and not necessarily the absence of an appropriate supervisor. However, approximately one in five interns felt that supervision was inadequate, particularly in PHC. Interns have also reported inadequate supervision and orientation from North West and Gauteng provinces.^17^ In this study, about a quarter of interns reported that orientation could have been better. There was also a need for more structured teaching programmes and building supervision capacity among medical officers.

Programme staff reported that while the interns required extra support and came with administrative requirements, they were always a valuable addition to the team and helped to improve service delivery. In the Western Cape, one of the health districts decided not to deploy interns, as they saw them as an unnecessary burden (personal communication). The results of this study should help to change such beliefs.

In general, it appears that in the Western Cape the overall aim of the new rotation ‘to produce a generalist doctor’ was met,^1^ namely someone who can function at a DH under supervision and independently in ambulatory care within the district health system. However, it should be noted that the emphasis is on a doctor with a generalist approach and not a ‘competent general practitioner’.^17^ This doctor should have an adequate approach to the undifferentiated patient, confidence in a broad range of DH clinical skills and experience in management of patients at PHC level. In the field of family medicine, there is a requirement for postgraduate training to ensure a competent general practitioner or family physician.^17^ Interns also reported more awareness of the discipline of family medicine and hopefully this will help the discipline reach its human resources for health goals.

### Strengths and limitations

As is common with surveys of this nature, there was a low response rate of only 21.8%, despite multiple attempts to reach and motivate the interns. A response rate of at least 30% would have allowed more accurate measurement (5% margin of error for 95% confidence intervals in a population of 330). Therefore, the generalisability of the results is limited by the response rate and the possibility of selection bias. It is not known if interns with a positive or negative experience were more likely to respond to the survey. Nevertheless, this is the first data to be collected on the new rotation and does provide some pointers to the strengths and weaknesses of the programme in the Western Cape.

The transferability of the results to other provinces should also be considered. The health services in the Western Cape may be better prepared for internship training. A lack of resources, medication and poorly functioning infrastructure have been reported by interns in other provinces.^17^ A study in KwaZulu-Natal reported that less than 10% of interns were satisfied with the conditions of the facilities and infrastructure, which influenced their perceptions of training and supervision.^18^ In addition, many other provinces have fewer family physicians to coordinate the training programmes and supervise.^18^

Some of the objectives of the HPCSA were not directly measured in the survey and questions could be added in the future. For example, on inter-professional teamwork, exposure to forensic medicine, mental health disorders and psychosocial problems.

The data were collected from the 2022 internship programme, which was disrupted to some extent by the coronavirus disease 2019 (COVID-19) pandemic. Interns may have been re-directed towards emergency and hospital-based care as well as testing for COVID-19, and other training opportunities may have been restricted.

### Recommendations and future research

Several recommendations can be made to strengthen the internship programme:

- Improve opportunities to engage with COPC, community health workers, community-based services, and public health medicine, through structured activities
- Enhance opportunities to engage with palliative care
- Identify gaps in supervision, particularly in PHC, and ensure that appropriate supervisors are directly available. Medical officers should be prepared for their supervisory role with an understanding of the HPCSA outcomes and basic training skills. Attention should be given to the role that community service doctors are playing in supervision and to ensure that eligible supervisors are available
- Recognise gaps in orientation and ensure that curators and supervisors adequately prepare interns
- Ensure that each setting has an adequate teaching programme and consider the use of digital technology to provide opportunities across the different settings
- Where possible, ensure exposure to continuity of care between the interns and patients, particularly in PHC.

While supervisors thought that the HPCSA logbook provided guidance on the expected outcomes and training opportunities,^1^ about 40% of interns felt it did not provide adequate guidance and did not help them to assess progress. The HPCSA should engage with interns on how to revise the logbook to make it more practically relevant.

The tools developed for this survey in the Western Cape should allow other provinces to duplicate the evaluation. It is important to administer the survey to interns before they exit the rotation and to make use of meetings and other opportunities to improve the response rate.

## Conclusion

The new internship programme in family medicine and primary care fulfilled its main aim to better prepare doctors to work as generalists in their community service year and beyond. Most respondents wanted to keep the rotation at 6-months. Interns learnt how to manage undifferentiated and chronic conditions, as well as how to refer and coordinate care between levels of the health system. They developed more independence, confidence, and resilience. Programmes need to improve structured exposure to COPC and public health medicine, palliative and ongoing care. Attention is needed to ensure adequate supervision and orientation. A low response limits the generalisability of the results and similar surveys should be conducted in other provinces.
